# Acute stress impacts reaction times in older but not in young adults in a flanker task

**DOI:** 10.1038/s41598-023-44356-4

**Published:** 2023-10-17

**Authors:** Greta Mikneviciute, Jens Allaert, Matias M. Pulopulos, Rudi De Raedt, Matthias Kliegel, Nicola Ballhausen

**Affiliations:** 1 NCCR LIVES–Overcoming Vulnerability: Life Course Perspectives, Swiss National Centre of Competence in Research, University of Geneva, Geneva, Switzerland; 2 Centre for the Interdisciplinary Study of Gerontology and Vulnerability, University of Geneva, Boulevard du Pont d’Arve 28, 1205 Geneva, Switzerland; 3 Department of Experimental Clinical and Health Psychology, Ghent University, Ghent, Belgium; 4 Ghent Experimental Psychiatry Lab, Department of Head and Skin, Ghent University, University Hospital Ghent (UZ Ghent), Ghent, Belgium; 5 Department of Psychology, University of Geneva, Geneva, Switzerland; 6 Department of Developmental Psychology, Tilburg University, Tilburg, The Netherlands

**Keywords:** Human behaviour, Cognitive ageing, Stress and resilience

## Abstract

Acute psychosocial stress effects on inhibition have been investigated in young adults, but little is known about these effects in older adults. The present study investigated effects of the Trier Social Stress Test on cognitive inhibition (i.e., ability to ignore distracting information) using a cross-over (stress vs. control) design in healthy young (*N* = 50; 18–30 years; *M*_age_ = 23.06) versus older adults (*N* = 50; 65–84 years; *M*_age_ = 71.12). Cognitive inhibition was measured by a letter flanker task and psychophysiological measures (cortisol, heart rate, subjective stress) validated the stress induction. The results showed that while stress impaired overall accuracy across age groups and sessions, stress (vs. control) made older adults’ faster in session 1 and slower in session 2. Given that session 2 effects were likely confounded by practice effects, these results suggest that acute psychosocial stress improved older adults’ RTs on a novel flanker task but impaired RTs on a practiced flanker task. That is, the interaction between stress and learning effects might negatively affect response execution when testing older adults on flanker tasks. If confirmed by future research, these results might have important implications especially in settings where repeated cognitive testing is performed under acute stress.

## Introduction

We are witnessing an unprecedented growth in both the size and the proportion of older people in the population^1^ as well as psychosocial distress in our society^2^. A unique set of stressors such as personal and financial losses, chronic illnesses, cognitive impairment, and caregiver burden can be experienced in the context of aging^3^. While the link between *chronic* stress and cognitive aging has been widely studied^4–6^, the same does not hold for *acute* stress and cognition in late adulthood. For instance, there is abundant research consistently pointing towards detrimental effects of chronic stress on diverse cognitive abilities in older adults^7–10^ but the studies tackling short-term cognitive effects after acute stress exposure have been mostly conducted in young adults^11–13^. In the last decade, a growing body of research on cognitive aging has addressed the effects of acute stress on memory performance^14–16^, but only few studies have examined the effects of acute stress on executive functioning in older age^17–19^. Given the well-established contribution of executive processing to the functioning of complex daily life activities in late adulthood^20,21^, it is important to understand how acute stress might impact performance of older adults’ executive abilities. Moreover, executive control largely relies on the prefrontal cortex (PFC)^22–25^, whose structure, activity and connectivity undergo age-related changes as part of the brain aging observed during late adulthood^26–29^, which makes the question of how stress affects executive functioning relying on PFC areas even more relevant in older age.

The few existing aging studies showed that compared to non-stressful conditions, acute stress had no effects on executive functioning^17–19^ in older adults, and a recent meta-analysis corroborated this further^14^. These results are in clear contrast with the vast literature investigating the effects of acute stress on PFC-dependent cognition in young adults^30,31^. In a meta-analysis in young adults, stress has been found to impair working memory and cognitive flexibility, whereas mixed effects have been shown for inhibition^12^.

Inhibition can be generally defined as the ability to down-regulate (suppress or ignore) the processing of information that is irrelevant to the task at hand^32,33^. Therefore, research suggests that inhibition is one of the mechanisms by which the PFC implements executive control^34–36^. In general, the literature distinguishes between at least two types of inhibition: *response inhibition* refers to the ability to suppress a dominant motor response, whereas *cognitive inhibition* (also called interference control or resistance to distracter interference) refers to the ability to ignore distractors that are irrelevant for the task’s performance^33,37,38^. In young adults, stress appears to have opposing effects depending on the type of inhibition process, namely, a better performance on response inhibition (e.g., stop-signal and Go/No-Go tasks) and impaired cognitive inhibition (e.g., flanker task)^12^*.*

Interestingly, these two types of inhibition also appear to be differently affected by the aging process: while there seems to be a general age-related decline in response inhibition (as measured by performance on Go/No-Go and stop-signal tasks), cognitive inhibition (as measured by performance on flanker and Stroop tasks) appears to be relatively intact when compared to young adults^39^. To the best of our knowledge, only one study so far investigated the effects of stress on inhibition in older men and focused exclusively on response inhibition^40^. This study showed that acute stress enhanced response inhibition accuracy in No-Go trials similarly in young and older men^40^. While this result is in line with a the meta-analytical findings on acute stress effects on response inhibition in young adults^12^, we are not aware of any study investigating the effects of acute stress on cognitive inhibition in older age.

Based on previous literature, acute stress could affect older adults’ cognitive inhibition in various ways. Enhanced performance under stress would be in line with a previous study in older adults on a different (i.e., response) inhibition task^40^. However, given that acute stress enhanced response inhibition but impaired cognitive inhibition in young adults^12^, impaired performance in older adults would further corroborate and extend meta-analytical findings in young adults indicating opposing effects of stress on response versus cognitive inhibition. Finally, considering that inhibition type also seems to differ in aging^39^ and given the overall absence of stress effects on executive functions in previous research in older adults^12^, no effects could be equally plausible.

In sum, given the crucial contribution of inhibitory processes in the executive control of thoughts and behaviors, it is important to understand how acute stress and aging interact in the context of inhibition. Moreover, further research using mixed-sex samples is warranted given that sex is a known moderating factor in stress and cognition research^16,41^. The goal of the present study is to fill the research gap regarding acute stress effects on cognitive inhibition (using a flanker task) in older age and contribute to the question of whether stress effects on cognitive inhibition differ between young and older adults. It was predicted that stress (vs. control) would impair cognitive inhibition performance in young adults^12^, while it was expected that older adults’ inhibition performance would be less affected by stress (vs. control), or to a lesser degree compared to young adults, based on null findings on executive functioning in late adulthood^14^ and the effects of aging on cognitive inhibition^39^.

## Method

### Design and participants 

The study used a between-subject (younger vs. older) cross-over (stress vs. control) design. Participants either started with the stress or control condition in a counterbalanced order (randomly assigned). At the second testing time (2–3 weeks later), they performed the respective other condition. Data collection took place from October 2020 to April 2022 (see Supplementary Materials S1). The study was conducted in accordance with the Swiss Federal Act on Research involving Human Beings and approved by the Cantonal Ethics Committee of Geneva. Participants received 100 CHF for their participation.

Hundred and six participants were recruited through advertisements in the Geneva community. Younger adults were mostly students across multiple disciplines. Older adults were community-dwelling older adults. Five older adults voluntarily dropped out of the study and an additional one was excluded a posteriori due to a health condition. Participants were included if they were 18–30 years or ≥ 65 years old and retired, healthy, and French-speaking. Exclusion criteria included prior experience with the Trier Social Stress Test (TSST, see description below)^42^, general anesthesia in the past three months, body mass index > 35, pregnant or lactating women, use of hormonal contraceptives or hormone replacement therapy, complete hysterectomy, diabetes, non-corrected visual or hearing problems, psychiatric diagnosis, drug abuse, presence of a stressful life event during the past 3 months. Given the high prevalence of hypertension in older adults^43^, anti-hypertensive or preventive medication in this age group was allowed (except for beta-blockers)^44^. Any psychiatric medication was not allowed. In addition, for older adults, the French version of the modified Telephone Interview of Cognitive Status (F-TICS-m)^45^ was used as a screening instrument for cognitive impairments. The F-TICS included questions regarding temporal and spatial orientation, recall of word lists to evaluate memory, questions evaluating semantic memory, and language. The maximum total score was 43; in line with the cut-off provided by the test authors, only participants who scored > 27 on the F-TICS-m were included in the study.

The sample size was a priori determined for a mixed ANOVA (within-between interaction) with G-Power 3.1 for a medium-to-large effect size (*N* = 90, *f*^2^ = 0.25, power ≥ 0.95 and α = 0.05). The final sample was composed of 100 healthy participants, 50 younger (50% F; 18–30 years; *M* = 23.06; *SD* = 2.90) and 50 older adults (56% F; 65–84 years; *M* = 71.12; *SD* = 5.02). Further sociodemographic information is presented in Tables 1 and S2. The two age groups did not differ in years of education (Table 1). However, older (vs. young) adults reported a higher number of medication intake, whereas young (vs. older) adults reported more perceived stress and depressive symptoms in the 2 weeks prior to testing (Table 1). Importantly, the two groups of participants within the same age group starting with either stress or control condition neither differed in mean age, years of education, medication intake, depression score, nor perceived stress score (all *p*s ≥ 0.078, see Table 2).

**Table 1:** Descriptive statistics (mean ± SD) and age differences of young and older adults.

Variables	Young adults (*N* = 50; 25 F) *M* (*SD*)	Older adults (*N* = 50; 28 F) *M* (*SD*)	Welch’s *t*	df	*p*
Age (years)	23.06 (2.90)	71.12 (5.02)	58.56	78.26	< .001
Education (years)	17.52 (2.23)	16.46 (4.53)	− 1.48	71.48	.142
N° medication	0.06 (0.24)	1.06 (1.39)	5.01	51.91	< .001
GDS	4.32 (3.66)	1.68 (2.09)	− 4.43	77.96	< .001
PSS-10	15.52 (7.03)	11.84 (5.42)	− 2.93	92.04	.004

H_a_ μ _0_ ≠ μ ^1^, For all variables, Levene’s test was significant (*p* < .001), suggesting a violation of the equal variance assumption. Therefore, Welch’s *t*-tests were used for all comparisons between age groups.

*GDS* Geriatric Depression Scale, *PSS-10* Perceived Stress Scale 10-item version.

**Table 2:** Independent samples T-test comparing participants starting with the stress versus control condition by age group.

Variables	Young adults (*N* = 25 vs. *N* = 25)				Older adults (*N* = 25 vs. *N* = 25)			
	Starting condition				Starting condition			
	Stress *M *(*SD*)	Control *M *(*SD*)	Mann–Whitney *U*	*p*	Stress *M *(*SD*)	Control *M *(*SD*)	Mann–Whitney *U*	*p*
Age (years)	22.64 (2.23)	23.40 (3.35)	286.50	.618	71.48 (5.08)	70.52 (4.86)	273.5	.454
Education (years)	17.44 (2.02)	17.60 (2.47)	307.00	.922	15.24 (4.49)	17.68 (4.32)	221.5	.078
N° medication	0.08 (0.28)	0.04 (0.20)	300.00	.571	1.16 (1.57)	0.96 (1.21)	308.00	.933
GDS	3.88 (3.64)	4.76 (3.70)	265.00	.358	1.48 (2.22)	1.88 (1.99)	241.50	.157
PSS-10	13.88 (7.07)	17.16 (6.73)	228.50	.104	11.04 (5.50)	12.64 (5.32)	258.50	.298

H_a_ μ 0 ≠ μ 1, For all variables, Shapiro–Wilk’s test was significant (*p* ≤ .015), suggesting a violation of the normality assumption. Therefore, Mann–Whitney-U t-tests were used for all comparisons.

*GDS* Geriatric Depression Scale, *PSS-10* Perceived Stress Scale 10-item version.

### Procedure

Potential participants completed an online screening with questions relating to the inclusion and exclusion criteria (see above) to verify their eligibility, and, at a second stage, eligible older adults were administered the F-TICS-m over the phone.

**Figure 1:**
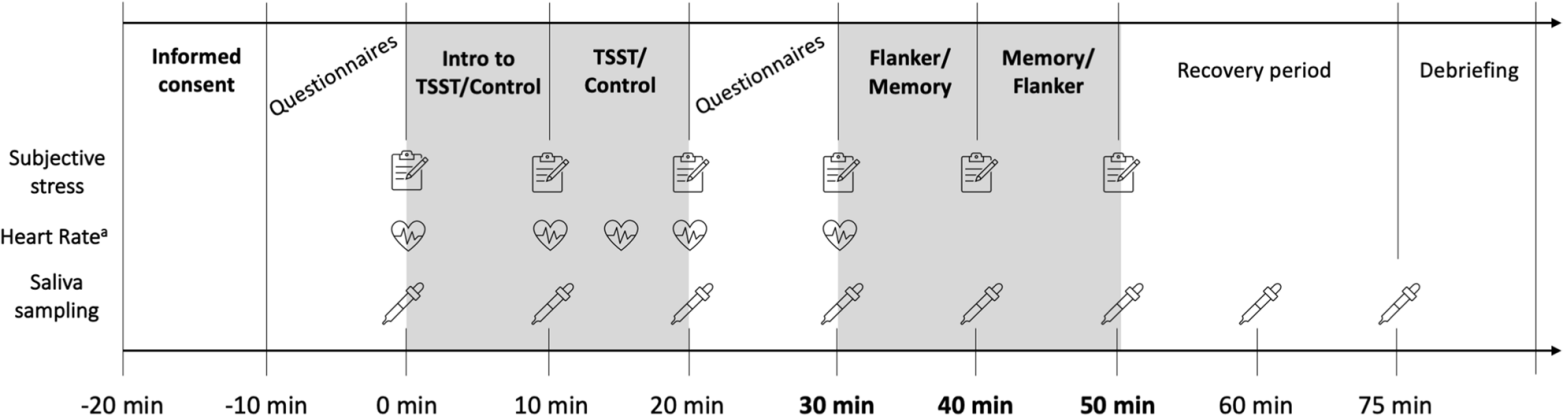
Exemplary timeline of the laboratory session. *TSST* Trier Social Stress Test. ^a^Heart rate epochs: 5–0 min (baseline); 5–10 min (anticipation); 10–15 min (TSST/control induction); 21–26 min (+ 1 min post-TSST); 26–31 min (+ 10 min post-TSST).

Figure 1 shows a timeline of the two laboratory sessions that took place either 14 h-16 h or 16 h-18 h depending on participants’ availability. Before each testing session, participants had to refrain from intense physical activity (48 h before) and physical activity during the day of the session, and alcohol consumption (24 h before). In addition, 1 h before the laboratory session, participants had to refrain from brushing their teeth and/or using dental floss, eating, or drinking (except water), smoking, or consuming stimulants (e.g., caffeine) as part of the upcoming salivary cortisol sampling procedure^46^.

Upon arrival, participants signed the informed consent and inclusion criteria specific to saliva sampling (see above) were verified by the experimenter. Additionally, the heart rate (HR) bracelet was put on the upper left arm of the participants. Then, participants filled out the demographic questionnaire (see Supplementary Materials S2 for complete information) and (when applicable) the menstrual/menopausal questionnaires. Next, participants provided the baseline samples for salivary cortisol, HR, and subjective stress measures. They were then introduced to the TSST/control instructions and received 5 min to prepare their speech before giving the oral presentation in front of the TSST jury/alone. At + 10 min and + 20 min after the TSST/control, the participants performed the flanker task and a memory retrieval task (results not presented here) in a counterbalanced order (see Fig. 1).

#### Experimental induction of acute stress 

Acute social stress was induced using the TSST^42^, which consists of three phases of five min each: preparation, oral presentation, and mental arithmetic task (i.e., 2043 – 17 – 17-…). Importantly, the script of the oral presentation was adapted for older adults. Instead of imagining applying for their dream job (i.e., instructions for younger adults), older adults had to provide argumentation for being the best candidate for a volunteering job at a club or charity group implicated in a subject that was dear to their hearts. Since the TSST was initially developed for use with younger adults, adapting the content of the oral presentation to a context that is relevant, hence valid, to older adults has been shown to successfully induce stress in older adults and is in line with previous studies using the TSST with this population^17,18,47^. The speech and arithmetic tasks took place in front of a camera and a jury of two experimenters (one woman, one man) wearing lab coats and keeping a strictly neutral demeanor toward the participant.

The so-called “placebo” version of the TSST was used as a validated control condition^48^. It is similar to the TSST but lacks the stressful features of the protocol (i.e., no camera, no jury). Additionally, instead of the mock job interview, the participant presents a book, trip, or movie of their choice alone in the room. The arithmetic task is replaced by simple additions (i.e., 15 + 15 + 15).

### Measures

#### Inhibition 

The flanker task^49^ was used to assess the ability to suppress responses that are inappropriate in a particular context. Accuracy and reaction times (RTs) for correct responses were used as main outcomes of the task. Participants were presented a 5-letter string in two versions counterbalanced between the visits: half of the participants started with the “KKCKK” version and participants with an even subject number started with the “HHSHH” version, performing the remaining condition at the second visit. Participants were asked to press a blue key if the central letter of the string was a K (or H), and a red key if it was a C (or S). Colored stickers were used to indicate the blue and red keys using the keys “P” and “Q” of the keyboard. Participants were instructed to answer as quickly and as accurately as possible to the target stimulus (i.e., central letter of the 5-letter string). The non-target stimuli flanking the target letter corresponded either to the same response as the target (i.e., congruent trials, e.g., CCCCC or KKKKK, 50% of trials), or to the opposite response (i.e., incongruent trials, e.g., KKCKK or CCKCC). There were 2 blocks of 40 trials per condition, for a total of 160 trials per participant. Each trial started with a central fixation cross (random interval between 500 and 1000 ms), followed by the presentation of the 5-letter string (maximum 3000 ms), and ended with a blank screen (1000 ms). The task was self-paced meaning that the participant’s keypress would trigger the presentation of the blank screen. The task was programmed with OpenSesame version 3.3.6^50^.

#### Salivary cortisol

Saliva samples were collected using Salivettes (Sarstedt, Switzerland). Participants had to keep a cotton swab in their mouths for two minutes and then store the swab in a plastic tube. Saliva samples were frozen and stored at − 20 °C until analysis. Salivary concentrations were measured using commercially available chemiluminescence immunoassay with high sensitivity (IBL International, Hamburg, Germany). The intra and interassay coefficients for cortisol were both below 9%. Each participant contributed with 8 samples per condition (see Fig. 1 for detailed timings).

#### Heart rate

HR was continuously monitored throughout the laboratory session starting at − 10 min using Scosche Rhythm24™, a wearable HR bracelet, and recorded using an iOS app (Heart Rate Variability Logger, Marco Altini). Average HR was calculated in epochs of 5 min for each stress-relevant phase (see Fig. 1 for further details).

#### Subjective stress

Subjective stress was measured using a single-item visual analogue scale question asking participants how stressed they were feeling at that moment on a continuum from 0 to 100 with high scores representing higher levels of subjective stress. The assessment was repeated six times per condition (see Fig. 1 for detailed timings).

### Data processing and statistical analyses 

RTs from incorrect and anticipatory responses (i.e., RTs < 250 ms) as well as RTs falling 2.5 *SD* beyond an individual’s mean per condition were removed^33^ from RTs analyses. One older participant was excluded from RT analyses because their mean RT in the stress condition was > 4 *SD* (*M* = 1207.47) from the mean RT of that same age group and condition (*M* = 612.35). Moreover, anticipatory (i.e., RTs < 250 ms) and late (> 1800 ms) responses were removed from accuracy analyses.

(Generalized) linear mixed models (GLMMs) were used to verify stress induction effectiveness on stress measures (i.e., manipulation checks, see results below) as well as to investigate the effects of stress on inhibition accuracy and RTs. LMMs involving manipulation checks (i.e., cortisol, HR, and subjective stress) included condition (stress, control), age (young, older), sex (male, female), and time (see Fig. 1 for timepoints) as fixed factors, and subject as random intercept. A GLMM of the binomial family with a logit link was employed for accuracy as dependent variable^51^, and a GLMM of the inverse Gaussian family was employed for RTs as dependent variable. Both GLMMs involving inhibition performance (i.e., accuracy and RTs) featured condition (stress, control), age (young, older), congruency (congruent, incongruent), and session (1, 2) as fixed factors, and subject as random intercept. Adding sex as a fixed factor did not improve the fit of the models and did not change the main results, thus, sex was omitted from analyses of cognitive measures to preserve statistical power. Finally, AIC and BIC indicators showed that adding a random slope by congruency in accuracy analysis and a random slope by condition in RT analysis improved the fit of the models (both *p*s ≤ 0.022), without changing any of the observed effects. Thus, the results presented below are based on those models (see Supplementary Materials S4 and S5).

### Author note

We thank Alexia Alfaro, Alexis Armellini, Maroussia Buchschacher, Christian Dal Busco, Nuriye Inan, Léa Marecaille, and Sinthujah Rasakumar for assistance with participants’ recruitment and data collection.

## Results

### Flanker measures 

Descriptive statistics and boxplots for both accuracy and RTs split by session, condition, age group, sex, and congruency can be found in Supplementary Materials S3.

#### Accuracy 

The GLMM for accuracy data showed a main effect of all factors: *condition* [*χ*^2^(1, *N* = 100) = 9.34, *p* = 0.002], *age* [*χ*^2^(1, *N* = 100) = 20.20, *p* < 0.001], *congruency* [*χ*^2^(1, *N* = 100) = 67.4747, *p* < 0.001], and *session* [*χ*^2^(1, *N* = 100) = 4.43, *p* = 0.035]. The main effects of *condition* and *age group* indicated that, overall, stress (*M* = 0.977, *SE* = 0.002) versus control (*M* = 0.984, *SE* = 0.002) impaired accuracy and that, overall, older adults (*M* = 0.99, *SE* = 0.002), were more accurate than young adults (*M* = 0.97, *SE* = 0.003). The main effect of *congruency* indicated that the task effectively induced inhibition costs, as seen by significantly lower accuracy in incongruent trials (*M* = 0.96, *SE* = 0.004) than in congruent trials (*M* = 0.99, *SE* = 0.001). Finally, the main effect of session indicated that, overall, participants were more accurate in session 1 (*M* = 0.984, *SE* = 0.002) versus session 2 (*M* = 0.978, *SE* = 0.002). No interaction was significant between these factors (all *p*s ≥ 0.08, see S4B).

#### Reaction times 

The GLMM for RTs data showed significant main effects of all factors: *condition* [*χ*^2^(1, *N* = 99) = 5.80, *p* = 0.016], *age* [*χ*^2^(1, *N* = 99) = 438.73, *p* < 0.001], *congruency* [*χ*^2^(1, *N* = 99) = 1173.63, *p* < 0.001], and *session* [*χ*^2^(1, *N* = 99) = 63.92, *p* < 0.001]. The main effect of *congruency* indicated that the task effectively induced inhibition costs, as seen by significantly slower RTs in incongruent trials (*M* = 549.27, *SE* = 3.23) than in congruent trials (*M* = 496.68, *SE* = 3.17). However, *congruency* did not interact with *condition* [*χ*^2^(1, *N* = 99) = 1.12, *p* = 0.291], reflecting that the inhibition cost did not differ between stress and control conditions. In addition, no higher-order interaction involving both *congruency* and *condition* was significant (all *p*s > 0.210, see Supplementary Materials S5B). Furthermore, the stress effect on overall RTs (i.e., averaged across congruency) differed between young and older adults across sessions [*condition x age x session, χ*^2^(1, *N* = 99) = 7.24, *p* = 0.007], and two related lower-order interactions were significant (both *p*s < 0.006), while *condition x age* was not (*p* > 0.20).

**Figure 2:**
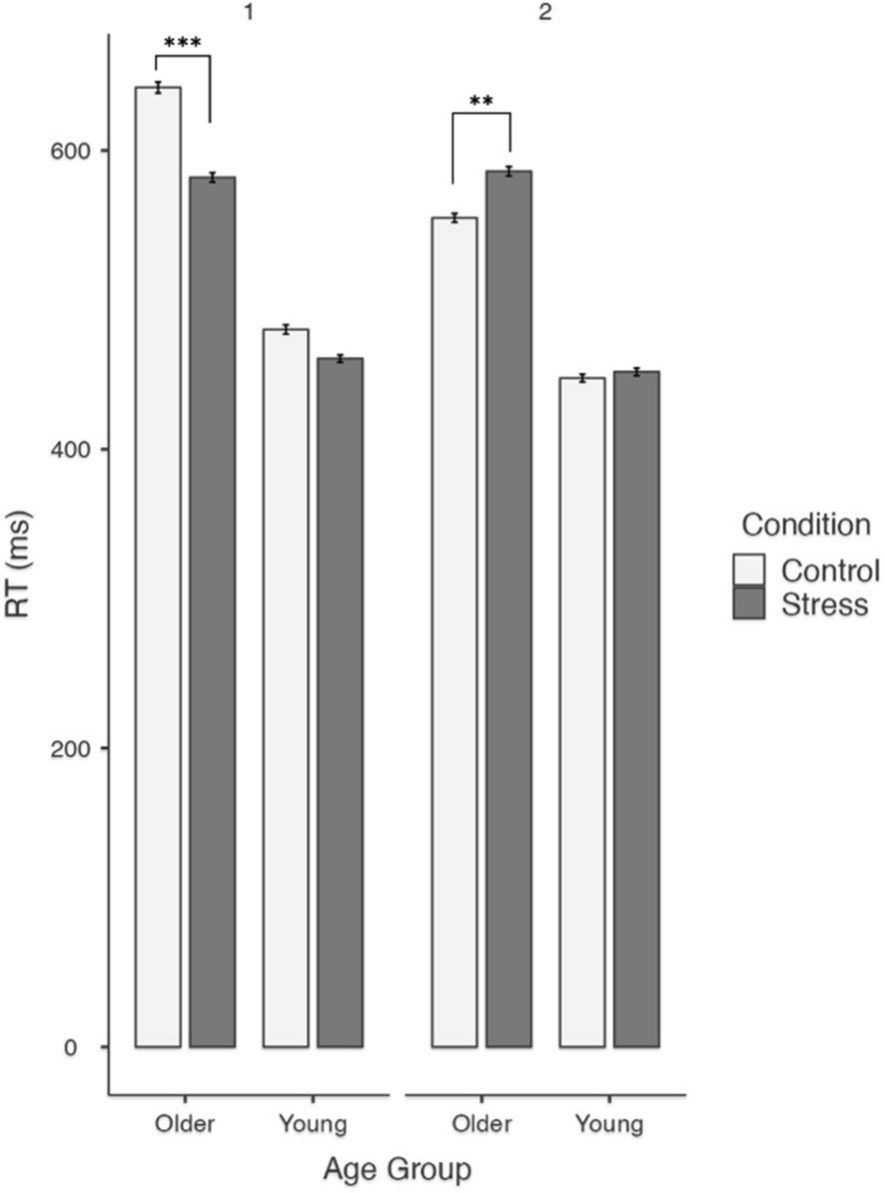
Stress Effects on RTs in young versus Older adults during Session 1 (1) versus Session 2 (2). Error bars represent SE.

The decomposition of *condition x age x session* showed that, during session 1, older adults were faster in the stress (vs. control) condition (*b* = − 57.81, *SE* = 10.50, *z* = − 5.51, *p* < 0.001), whereas the effect was inversed during session 2, with older adults being slower in the stress (vs. control) condition (*b* = 30.55, *SE* = 10.34, *z* = 2.95, *p* = 0.003, Fig. 2). In contrast, in young adults, stress (vs. control) did not affect RTs in either session (both *p*s > 0.12, Fig. 2). Finally, the model showed an *age x congruency* interaction [*χ*^2^(1, *N* = 99) = 41.55, *p* < 0.001]. Follow-up analysis revealed that, averaged across conditions and sessions, the inhibition cost of RTs (i.e., congruent—incongruent trials) was overall greater for older compared to young adults (*b* = − 19.80, *SE* = 3.07, *z* = − 6.44, *p* < 0.001). None of the remaining effects reached significance (all *p*s > 0.420, see S5B).

### Manipulation checks 

Detailed results regarding age differences on the manipulation check variables (i.e., cortisol, HR, subjective stress) were published elsewhere^52^. Here, only results that are relevant to demonstrate an effective stress induction (i.e., significant increase in stress vs. control within each age group for each variable) are presented.

#### Salivary cortisol 

The LMM predicting cortisol levels revealed significant main effects of all factors: *condition* [*F*(1, 1493.06) = 285.31, *p* < 0.001], *age* [*F*(1, 100) = 15.86, *p* < 0.001], *sex* [*F*(1, 100) = 26.87, *p* < 0.001], and *time* [*F*(7, 1493.06) = 26.58, *p* < 0.001]. There was a significant *condition x age x time* interaction*, F*(7, 1493.03) = 2.35, *p* = 0.022), and all lower-order interactions were significant (*condition x age, condition x time, age x time*, all *p*s < 0.005, see S7). Importantly, in both age groups, cortisol levels were higher for participants in the stress (vs. control) condition starting from + 20 min until the end of the testing session (all *p*s ≤ 0.015, see S7).

#### Heart rate

There was a significant main effect of *condition* [*F*(1, 876.97) = 58.47, *p* < 0.001], *age* [*F*(1, 100.28) = 6.28, *p* = 0.014], and *time* [*F*(4, 872.38) = 82.90, *p* < 0.001], but not *sex* [*F*(1, 100.28) = 1.43, *p* = 0.235]. Moreover, *condition* x *age* [*F*(1, 876.97) = 13.38, *p* < 0.001], and *condition* x *time* [*F*(4, 872.38) = 12.19, *p* < 0.001] interactions were significant. Both age groups showed higher HR in the stress (vs. control) condition [younger:* b* = 5.21, *SE* = 0.66, *t*(879.42) = 7.90, *p* < 0.001; older: *b* = 1.84, *SE* = 0.64, *t*(874.37) = 2.85, *p* = 0.004]. Although HR did not differ between stress versus control conditions at baseline [*b* = − 0.58, *SE* = 1.02, *t*(873.32) = − 0.56,* p* = 0.574], HR was higher in the stress (vs. control) condition in the anticipation, TSST, and immediate post-TSST phase (all *p*s < 0.005), but was again comparable between conditions + 10 min post TSST versus control [*b* = 1.91, *SE* = 1.02, *t*(873.30) = 1.87,* p* = 0.062, see S8].

#### Subjective stress

There were significant main effects of *condition* [*F*(1, 1100) = 98.92, *p* < 0.001], and *time* [*F*(5, 1100) = 16.54, *p* < 0.001], but not *age* [*F*(1, 100) = 0.60, *p* = 0.441], nor *sex* [*F*(1, 100) = 0.23, *p* = 0.630]. Furthermore, *condition* interacted with *age* [*F*(1, 1100) = 5.23, *p* = 0.022], and *time* [*F*(5, 1100) = 11.50, *p* < 0.001]. Follow-up analysis of *condition x age* showed that both age groups reported more stress in the stress (vs. control) condition [younger: *b* = 9.83, *SE* = 1.14, *t*(1100) = 8.65, *p* < 0.001; older: *b* = 6.15, *SE* = 1.14, *t*(1100) = 5.42, *p* < 0.001]. The difference between conditions was larger in younger than older adults [*b* = 3.67, *SE* = 1.62, *t*(1117) = 2.27,* p* = 0.023]. No age differences were found in subjective stress in the control condition [*b* = 0.66, *SE* = 3.49, *t*(111.49) = 0.19,* p* = 0.850] nor in the stress condition [*b* = 4.34, *SE* = 3.49, *t*(111.49) = 1.24,* p* = 0.216]. *Condition x time* interaction showed that while subjective stress did not differ between the stress and control conditions at baseline [*b* = − 0.24, *SE* = 1.97, *t*(1100) = − 0.12,* p* = 0.903], more stress was present in the stress (vs. control) condition from + 10 to + 40 min (all *p*s < 0.015), but was again comparable between conditions by + 50 min [*b* = 2.98, *SE* = 1.97, *t*(1100) = 1.51, *p* = 0.130, see S9].

## Discussion 

Using a between-subjects (young vs. older) cross-over (stress vs. control) design, the present study was the first to investigate possible age differences in acute stress effects on a flanker task measuring cognitive inhibition. The results indicated that the experimental manipulation successfully induced stress in both age groups, as measured by significant increases in cortisol, HR, and subjective stress levels in the stress (vs. control) condition. Moreover, the presence of a congruency effect for both accuracy and RTs reflected that the flanker task was effective in inducing inhibition costs (i.e., reduced performance for incongruent vs. congruent trials) associated with the cognitive effort of ignoring the distracting stimuli during incongruent (vs. congruent) trials. However, congruency did not interact with condition neither for accuracy nor RTs, meaning that the inhibition cost was not affected by stress. Given that the main research question focused on stress effects on inhibition performance best represented by a change in inhibition costs, the absence of an interaction between congruency and condition in both accuracy and RTs results suggested that stress induced a more general effect on RTs in a flanker inhibition task that is not specific to inhibiting distractors in incongruent trials.

The analyses showed that, overall, stress impaired flanker’s accuracy regardless of age. The direction of the main (negative) effect is in line with our predictions and with a previous meta-analysis in young adults^12^. Of note, however, the session effect showed that, overall, participants were more accurate during session 1 than session 2. Moreover, stress affected flanker’s RTs depending on age group and session: averaged over congruency, stress made older adults *faster* during session 1 and *slower* during session 2, whereas young adults’ RTs remained unaffected by the stress induction. Thus, concerning age differences, in the present study stress impaired overall flanker’s accuracy across age groups but affected only older adults’ response execution, improving flanker’s RTs in session 1 and impairing performance in session 2.

However, the interpretation of session 2 RT results must consider that stress effects were confounded by the session effect, which could be explained by an underlying interaction between stress and practice effects on cognitive performance. Although stress effects on learning were beyond the scope of this study, we hereby attempt a potential explanation accounting for the opposite pattern in session 1 versus session 2 in older adults. In fact, when looking at the existing literature of stress effects on cognition, it is not uncommon to observe reversed effects of stress once the task is practiced or well-rehearsed^41^. Given the established impairing effects of stress on memory retrieval^11,53^, one potential mechanism explaining how practicing a task could reverse stress effects on said task might be that the retrieval of specific task strategies that were learned during session 1 under control conditions might be impaired in session 2 under stress conditions^41^. The interaction between condition, age group, and session in RTs suggests that, despite counterbalancing the starting condition, stress impacted not only session 1 versus session 2, but also young versus older adults RTs differently. Interestingly, as shown by the absence of session effects in young adults, older adults appeared to be more negatively impacted by the interaction between stress and session (or practice effects). This is in line with a previous study showing an effect of acute stress on learning after interference in older but not in young adults^54^.This is in line with a previous study showing an effect of acute stress on learning after interference in older but not in young adults^54^. If confirmed by future research, these results might have important implications in how we conceptualize the dynamic relationship between stress and cognition in older age and might be especially relevant in clinical settings where repeated cognitive testing is performed.

Moreover, when comparing stress (vs. control) effects in session 1 versus 2, it’s important to consider that the participants in the control condition of session 1 were new to the task whereas the participants in the control condition of session 2 had previously learned the task under stressful conditions. In other words, enhancing effects of stress are more likely to be observed during session 1 than session 2, not only because in session 2 stress might impair retrieval of previously learned task strategies but also because in session 2 it is more difficult to outperform a control condition where participants have already rehearsed the task under stressful conditions. In addition, given that a moderate amount of stress is known to be beneficial for learning and skill consolidation^55^, it might be argued that participants who are first exposed to the task under stressful conditions are better able to learn and consolidate task strategies, which might carry a beneficial effect for performance under control conditions during session 2. Therefore, the control condition of session 1 is not equivalent to the control condition of session 2, which might result in paradoxical effects when stress is compared to control groups that differ in the practice levels of the task across different sessions, contributing to explain reversed stress effects on practiced tasks.

Taken together, the reversed pattern of results observed in older adults when comparing session 1 versus 2 could be explained by a complex interaction between stress and practice effects susceptible to enhance not only (short-term) cognitive performance when the task is first encountered under stress conditions but also subsequent (long-term) cognitive performance under control conditions in a cross-over design such as in the present study. Moreover, this limits the possible conclusions that can be drawn from session 2 regarding stress effects on cognitive inhibition in older adults, as the current design did not test conditions of stress/control at both session 1 and 2. In addition, although null effects of stress in young adults could be explained by the task being too easy for them, the results seem to hint towards potential age differences in the way stress interacts with practice effects. Thus, the possibility that stress may interact with practice effects differently across the lifespan is important to consider, and directly test, in future studies investigating stress and cognition in developmental populations using within-subject designs.

### Potential mechanisms underlying stress effects on cognitive performance and future perspectives

A promising venue for future research is replicating the present and previous^40^ effects of stress on inhibition in young and older adults using both a response and a cognitive inhibition task in the same design. This would allow to confirm meta-analytical findings in young adults suggesting that stress has opposing effects on response versus cognitive inhibition, which, to the best of our knowledge, have not yet been formally tested in the same research design. In addition, this would also allow to directly compare young and older adults’ stress effects on both inhibition processes, thus advancing knowledge on possible age differences in the cognitive effects of acute stress.

In young adults, stress is thought to induce a shift commonly called “from thinking to doing”, i.e., from top-down goal-directed behavior towards bottom-up resource-saving habitual behavior^30,56,57^. In this sense, much like the fight-or-flight response aims at reallocating bodily energy resources to physically fight or flight from the stressor^58^, the cognitive effects of stress are also thought to reallocate limited executive resources to efficiently cope with the stressor^30,56,57^. This would explain why some cognitive functions seem to benefit from stress (e.g., risk-taking, memory retention), while others are impaired (e.g., memory recall, goal-directed behavior)^41,59,60^. Research in young adults^61^ showed that acute stress can indeed selectively improve control over motor actions without affecting accuracy nor inhibition costs in a flanker task, which is in line with the present results observed in older adults. It has been suggested that by impairing executive control over thoughts (e.g., cognitive inhibition) but improving executive control over motor actions (e.g., response inhibition), the cognitive effects of stress would facilitate either fleeing from or fighting with the current stressor^12^. This might provide a potential explanation as to why older adults are faster without necessarily being more accurate in their executive performance. Nevertheless, our study did not evaluate outcomes associated with response inhibition, which is the type of inhibition that previous studies have shown to be improved by stress^12,40,62^. Thus, it remains unclear whether the enhancing effect of stress on the execution of motor actions arises from the same underlying mechanism as the effects of stress on response inhibition^12^. Moreover, if that would be the case, the results from the present study suggest that this effect might be further modulated by practice, at least concerning older adults.

Moreover, the idea of a stress-induced shift in cognition lacks a developmental perspective and it would be valuable to inquire whether the shift from “thinking to doing” might be susceptible to change and evolve over the lifespan as a result of aging processes. For instance, the stress-induced shift in cognition in late adulthood might be shaped by a combination of physiological aging and psychological experience. Older adults may employ different strategies to deal with stress to compensate for the loss of bodily strength and sensory processing. Future research would significantly contribute to advance both the stress and the cognitive aging fields by integrating a developmental perspective over the stress-induced shift on cognition and by formally testing these hypotheses through experimental studies comparing cognitive performance of different age groups under acute stress. Moreover, future research should explore the connection between developmental time periods and individual susceptibility to the environment (thus stress conditions), taking into account variations in susceptibility both within and across individuals over time (see differential susceptibility theory^63^). It is important to investigate whether individuals who are highly or minimally influenced by the environment, especially specific environmental factors such as stressors, during early stages of life maintain their levels of malleability in late adulthood^63^.

### Limitations 

A limitation of the present study is the timeline of data collection which overlapped with the COVID-19 pandemic. Age differences at baseline in self-reported chronic stress (i.e., Perceived Stress Scale) and depressive symptoms (i.e., Geriatric Depression Scale) were assessed, showing that young adults in the present study reported significantly more stress and depression symptoms than older adults (Table 1). This is line with recent reports showing that young adults’ mental health was more negatively affected by the COVID-19 pandemic than older adults’ mental health^64,65^. Thus, we cannot rule out the possibility that baseline differences in chronic stress and depression might explain the age differences in stress effects observed in the present study. Although these differences would not invalidate the conclusions made above regarding older adults’ performance, it could represent an alternative explanation to the null findings in young adults. This might also explain why we failed at replicating previous results on stress and cognitive inhibition in young adults. Thus, it is important to replicate the present results in both younger and older adults in a non-pandemic context.

Moreover, another important limitation is that the lack of interaction between condition and congruency prevents us from concluding about stress effects on inhibition performance specifically. Rather, stress induced a more general effect on flanker’s RTs that is not specific to inhibiting distractors in incongruent trials. Additionally, although the starting condition was randomized between participants, we cannot exclude the possibility of baseline differences in RTs between older adults who started with the stress versus control condition, despite the absence of differences on socio-demographical variables (see Table 2).

Furthermore, although sex was present as a predictor for analyses involving psychophysiological variables, sex was not included in the analyses involving cognitive variables, as the addition of a fifth fixed factor would have reduced power to detect the effects of interest, other than resulting in overly-complex statistical models. Given that the research questions focused on age differences and not sex differences, a simpler model was deemed sufficient to answer these research questions. However, this comes with the limitation that sex might have confounded RTs results. A bigger sample size is warranted in future studies not only to better account for potential interactions between stress, age, and sex on cognition but also, on a more basic level, to be able to detect smaller effects of stress on cognition. For example, our results showed a marginally significant interaction of stress with age suggesting that this effect might change with age. Future studies with even larger sample sizes are warranted to investigate if this would be indeed the case.

Finally, although two different validated versions of the flanker task (HS vs. KC letters) were used, confounding practice effects were present in RTs results. A within-subjects design was preferred because older participants are more difficult to recruit and they present higher interindividual variability in cognitive performance than young participants, which can be controlled for using a within-subjects design. However, findings of the present study provide additional evidence that especially in stress and cognition research, studies using between-subjects designs can show substantially different results from studies that use within-subjects designs to test the cognitive effects of stress^41^. Unless the researcher is specifically interested in the effects of stress on learning or practice effects, to avoid confounding effects of practice we would advise future research on stress and cognitive aging to prefer well-powered between-subject designs.

## Conclusion 

The present study was the first to examine possible age differences in acute stress effects on cognitive inhibition in a sample of young and older adults using a within-subjects design. While stress impaired overall accuracy across age groups, the results showed novel findings indicating that stress improved RTs in an unpracticed flanker task but impaired RTs in a practiced flanker task in older adults only. The present results represent an important contribution in the field of stress and cognitive aging showing that acute stress may have the ability to improve older adult’s RTs. However, stress and learning effects might negatively affect RTs when testing older adults on practiced tasks. If confirmed by future research, these results might have important clinical implications especially in settings where repeated cognitive testing is performed.

### Supplementary Information

Supplementary Information.

## Data Availability

The cognitive dataset analyzed during the current study is publicly available on: https://osf.io/qchms/.
