# Supplementary material for: Acute stress impacts reaction times in older but not in young adults in a flanker task
Source: Sci Rep. 2023 Oct 17;13:17690. doi: 10.1038/s41598-023-44356-4 (PMC10582047; doi:10.1038/s41598-023-44356-4)
Supplement: Supplementary file 1 — Supplementary Information. [file 41598_2023_44356_MOESM1_ESM.docx]

Table of Contents

[S1. List of COVID19 sanitary measures applied during the testing sessions 2](#_Toc137130665)

[S2. Complete participants’ sociodemographic background 3](#_Toc137130666)

[S3. Descriptive statistics Flanker task performance 5](#_Toc137130667)

[S4. Flanker's accuracy model 10](#_Toc137130668)

[A. Accuracy model information 10](#_Toc137130669)

[B. Accuracy model results 11](#_Toc137130670)

[S5. Flanker's RTs model 12](#_Toc137130671)

[A. RTs model information 12](#_Toc137130672)

[B. RTs model results 13](#_Toc137130673)

[S6. Cortisol model 14](#_Toc137130674)

[S7. HR model 16](#_Toc137130675)

[S8. Subjective stress model 18](#_Toc137130676)

# List of COVID19 sanitary measures applied during the testing sessions

- Participants disinfect their hands upon arrival in the laboratory and all surfaces and electronic material are disinfected in between participants.
- The lead experimenter keeps 1.5 m with the participant whenever possible.
- The participant and the lead experimenter always wear a mask. An exception is made for the participant in the TSST part (see below).

TSST protocol

- The 2 TSST jurors arrive at the laboratory shortly before the start of the protocol and only stay in the room for the duration of the TSST (i.e., approximately 15 min).
- The participant is placed 4 meters away from the 2 jurors, who are separated by plexiglass.
- When the participant enters the room of the TSST, they are asked to take off their mask and the TSST jurors do the same. This is necessary because the mask would otherwise cover important facial cues and not being able to see the experimenters’ expressions could reduce the socio-evaluative threat component of the TSST. In addition, it might give a false sense of security to the participant during the TSST and could reduce the overall effectiveness of the protocol.
- As soon as the stress protocol is finished, the participant puts their mask back on and the 2 jurors leave the room.

Saliva samples

- Participants collect saliva by themselves, the experimenter guides them orally and at a distance through the procedure. The experimenter is never in contact with the participant’s saliva.
- The participant is instructed to lower their mask and let the cotton swab of the Salivette gently "fall" into their mouth. The participant keeps their mask on during the wait time. After 2 min, the participant recovers the cotton swab wearing a glove on their dominant hand and they are asked to carefully place the cotton with saliva from their mouth back in the Salivette's container. The glove is immediately disposed of. The participant is then asked to seal the Salivette and disinfect their hands.
- The experimenter wears a pair of gloves when placing the 8 collected Salivettes in a plastic zip bag to be stored in a freezer.

# Complete participants’ sociodemographic background

Sociodemographic characteristics of young (*N* = 50) and older participants (*N* = 50).

| Characteristic | Young | | Older | |
| --- | --- | --- | --- | --- |
|  | Men (*N* = 25) | Women (*N* = 25) | Men (*N* = 22) | Women (*N* = 28) |
| Age |  |  |  |  |
| *M* (*SD*) | 23.92 (3.11) | 22.20 (2.45) | 71.86 (5.96) | 70.54 (4.17) |
| Range | 18-30 | 19-30 | 65-84 | 65-77 |
| Sexual orientation |  |  |  |  |
| Heterosexual | 23 (92 %) | 23 (92 %) | 22 (100 %) | 28 (100 %) |
| Homosexual | 2 (8 %) | 0 (0 %) | 0 (0 %) | 0 (0 %) |
| Bisexual | 0 (0 %) | 2 (8 %) | 0 (0 %) | 0 (0 %) |
| Nationality |  |  |  |  |
| French | 2 (8 %) | 5 (20 %) | 1 (4.55 %) | 2 (7.14 %) |
| Swiss | 19 (76 %) | 17 (68 %) | 18 (81.82 %) | 25 (89.29 %) |
| Other | 4 (16 %) | 3 (12 %) | 3 (13.64 %) | 1 (3.57 %) |
| No answer | 0 (0 %) | 0 (0 %) | 0 (0 %) | 0 (0 %) |
| Marital status |  |  |  |  |
| Single | 14 (56 %) | 17 (68 %) | 2 (9.09 %) | 8 (28.57 %) |
| In couple | 8 (32 %) | 8 (32 %) | 2 (9.09 %) | 1 (3.57 %) |
| Married, remarried or PACS | 3 (12 %) | 0 (0 %) | 13 (59.09 %) | 8 (28.57 %) |
| Separated or divorced | 0 (0 %) | 0 (0 %) | 2 (9.09 %) | 8 (28.57 %) |
| Widow(er) | 0 (0 %) | 0 (0 %) | 3 (13.64 %) | 3 (10.71 %) |
| Level of education |  |  |  |  |
| Primary school | 0 (0 %) | 0 (0 %) | 0 (0 %) | 0 (0 %) |
| Middle school | 0 (0 %) | 2 (8 %) | 0 (0 %) | 1 (3.57 %) |
| High school | 11 (44 %) | 13 (52 %) | 7 (31.82 %) | 11 (39.29 %) |
| University | 13 (52 %) | 10 (40 %) | 10 (45.45 %) | 11 (39.29 %) |
| Postgraduate | 1 (4 %) | 0 (0 %) | 5 (22.73 %) | 5 (17.86 %) |
| Professional activity |  |  |  |  |
| Student | 13 (52 %) | 13 (52 %) | 0 (0 %) | 0 (0 %) |
| Student-employee | 6 (24 %) | 10 (40 %) | 0 (0 %) | 0 (0 %) |
| Employee | 2 (8 %) | 2 (8 %) | 0 (0 %) | 0 (0 %) |
| Independent | 2 (8 %) | 0 (0 %) | 1 (4.55 %) | 0 (0 %) |
| Pensioner | 0 (0 %) | 0 (0 %) | 21 (95.45 %) | 28 (100 %) |
| Unemployed | 2 (8 %) | 0 (0 %) | 0 (0 %) | 0 (0 %) |
| Incapacity to work | 0 (0 %) | 0 (0 %) | 0 (0 %) | 0 (0 %) |
| Annual income (CHF) |  |  |  |  |
| 0 – 19 999 | 20 (80 %) | 21 (84 %) | 0 (0 %) | 2 (7.14 %) |
| 20 000 – 39 999 | 3 (12 %) | 3 (12 %) | 1 (4.55 %) | 3 (10.71 %) |
| 40 000 – 59 999 | 2 (8 %) | 0 (0 %) | 2 (9.09 %) | 8 (28.57 %) |
| 60 000 – 79 999 | 0 (0 %) | 1 (4 %) | 8 (36.36 %) | 9 (32.15 %) |
| 80 000 – 99 999 | 0 (0 %) | 0 (0 %) | 3 (13.64 %) | 3 (10.71 %) |
| 100 000 or more | 0 (0 %) | 0 (0 %) | 6 (27.27 %) | 2 (7.14 %) |
| No answer | 0 (0 %) | 0 (0 %) | 2 (9.09 %) | 1 (3.57 %) |
| Religion |  |  |  |  |
| Religious | 14 (56 %) | 13 (52 %) | 7 (31.82 %) | 14 (50 %) |
| Atheist | 7 (28 %) | 10 (40 %) | 8 (36.36 %) | 10 (35.71 %) |
| Agnostic | 4 (16 %) | 2 (8 %) | 7 (31.82 %) | 4 (14.29 %) |
| Vaccination COVID-19 |  |  |  |  |
| No | 0 (0 %) | 0 (0 %) | 1 (4.55 %) | 4 (14.29 %) |
| 1st Dose | 0 (0 %) | 0 (0 %) | 1 (4.55 %) | 0 (0 %) |
| 2nd Dose | 0 (0 %) | 0 (0 %) | 12 (54.55 %) | 17 (60.71 %) |
| No answer | 25 (100 %) | 25 (100 %) | 8 (36.36 %) | 7 (25 %) |
| Dietary |  |  |  |  |
| High-fat diet | 4 (16 %) | 4 (16 %) | 1 (4.55 %) | 0 (0 %) |
| Balanced diet | 17 (68 %) | 14 (56 %) | 18 (81.82 %) | 22 (78.57 %) |
| Low-fat diet | 3 (12 %) | 4 (16 %) | 2 (9.09 %) | 6 (21.43 %) |
| Vegetarian or Vegan | 1 (4 %) | 3 (12 %) | 1 (4.55 %) | 0 (0 %) |
| Smoker |  |  |  |  |
| No | 18 (72 %) | 23 (92 %) | 18 (81.82 %) | 25 (89.29 %) |
| Yes | 6 (24 %) | 2 (8 %) | 3 (3.64 %) | 3 (10.71 %) |
| No answer | 1 (4 %) | 0 (0 %) | 1 (4.55 %) | 0 (0 %) |
| Perceived Stress Scale (PSS) |  |  |  |  |
| *M* (*SD*) | 14.28 (7.17) | 16.76 (6.80) | 10.55 (4.53) | 12.64 (6.09) |
| Coronavirus Impact Scale (CIS) |  |  |  |  |
| *M* (*SD*) | 7.52 (3.39) | 8.44 (3.66) | 5.36 (3.16) | 5.07 (2.62) |
| Last mealtime Control (hours) |  |  |  |  |
| *M* (*SD*) | 11.31 (3.90) | 11.46 (3.19) | 11.76 (2.06) | 11.71 (1.90) |
| Last mealtime Stress (hours) |  |  |  |  |
| *M* (*SD*) | 10.95 (4.48) | 11.25 (3.87) | 12.05 (1.05) | 11.51 (1.24) |

# Descriptive statistics Flanker task performance

| Descriptive statistics of Flanker's accuracy by session, condition, age group, sex, and congruency. | | | | | | | | | | | | | | | | | | | |
| --- | --- | --- | --- | --- | --- | --- | --- | --- | --- | --- | --- | --- | --- | --- | --- | --- | --- | --- | --- |
|  | | **Session** | | **Condition** | | **Age Group** | | **Sex** | | **Congruency** | | **Mean** | | **SD** | | **Minimum** | | **Maximum** | |
| **Accuracy** |  | 1 |  | Control |  | Older |  | Female |  | Congruent |  | 1.00 |  | 0.06 |  | 0 |  | 1 |  |
|  |  |  |  |  |  |  |  |  |  | Incongruent |  | 0.97 |  | 0.16 |  | 0 |  | 1 |  |
|  |  |  |  |  |  |  |  | Male |  | Congruent |  | 1.00 |  | 0.05 |  | 0 |  | 1 |  |
|  |  |  |  |  |  |  |  |  |  | Incongruent |  | 0.97 |  | 0.16 |  | 0 |  | 1 |  |
|  |  |  |  |  |  | Young |  | Female |  | Congruent |  | 0.99 |  | 0.10 |  | 0 |  | 1 |  |
|  |  |  |  |  |  |  |  |  |  | Incongruent |  | 0.94 |  | 0.24 |  | 0 |  | 1 |  |
|  |  |  |  |  |  |  |  | Male |  | Congruent |  | 0.99 |  | 0.12 |  | 0 |  | 1 |  |
|  |  |  |  |  |  |  |  |  |  | Incongruent |  | 0.92 |  | 0.27 |  | 0 |  | 1 |  |
|  |  |  |  | Stress |  | Older |  | Female |  | Congruent |  | 0.99 |  | 0.12 |  | 0 |  | 1 |  |
|  |  |  |  |  |  |  |  |  |  | Incongruent |  | 0.96 |  | 0.20 |  | 0 |  | 1 |  |
|  |  |  |  |  |  |  |  | Male |  | Congruent |  | 0.99 |  | 0.10 |  | 0 |  | 1 |  |
|  |  |  |  |  |  |  |  |  |  | Incongruent |  | 0.98 |  | 0.14 |  | 0 |  | 1 |  |
|  |  |  |  |  |  | Young |  | Female |  | Congruent |  | 0.99 |  | 0.10 |  | 0 |  | 1 |  |
|  |  |  |  |  |  |  |  |  |  | Incongruent |  | 0.90 |  | 0.31 |  | 0 |  | 1 |  |
|  |  |  |  |  |  |  |  | Male |  | Congruent |  | 0.99 |  | 0.12 |  | 0 |  | 1 |  |
|  |  |  |  |  |  |  |  |  |  | Incongruent |  | 0.95 |  | 0.22 |  | 0 |  | 1 |  |
|  |  | 2 |  | Control |  | Older |  | Female |  | Congruent |  | 0.99 |  | 0.08 |  | 0 |  | 1 |  |
|  |  |  |  |  |  |  |  |  |  | Incongruent |  | 0.98 |  | 0.15 |  | 0 |  | 1 |  |
|  |  |  |  |  |  |  |  | Male |  | Congruent |  | 1.00 |  | 0.07 |  | 0 |  | 1 |  |
|  |  |  |  |  |  |  |  |  |  | Incongruent |  | 0.95 |  | 0.21 |  | 0 |  | 1 |  |
|  |  |  |  |  |  | Young |  | Female |  | Congruent |  | 0.99 |  | 0.12 |  | 0 |  | 1 |  |
|  |  |  |  |  |  |  |  |  |  | Incongruent |  | 0.90 |  | 0.30 |  | 0 |  | 1 |  |
| **Accuracy** |  | 2 |  | Control |  | Young |  | Male |  | Congruent |  | 0.98 |  | 0.12 |  | 0 |  | 1 |  |
|  |  |  |  |  |  |  |  |  |  | Incongruent |  | 0.95 |  | 0.22 |  | 0 |  | 1 |  |
|  |  |  |  | Stress |  | Older |  | Female |  | Congruent |  | 0.99 |  | 0.11 |  | 0 |  | 1 |  |
|  |  |  |  |  |  |  |  |  |  | Incongruent |  | 0.95 |  | 0.21 |  | 0 |  | 1 |  |
|  |  |  |  |  |  |  |  | Male |  | Congruent |  | 0.99 |  | 0.10 |  | 0 |  | 1 |  |
|  |  |  |  |  |  |  |  |  |  | Incongruent |  | 0.96 |  | 0.20 |  | 0 |  | 1 |  |
|  |  |  |  |  |  | Young |  | Female |  | Congruent |  | 0.98 |  | 0.14 |  | 0 |  | 1 |  |
|  |  |  |  |  |  |  |  |  |  | Incongruent |  | 0.94 |  | 0.24 |  | 0 |  | 1 |  |
|  |  |  |  |  |  |  |  | Male |  | Congruent |  | 0.97 |  | 0.17 |  | 0 |  | 1 |  |
|  |  |  |  |  |  |  |  |  |  | Incongruent |  | 0.90 |  | 0.31 |  | 0 |  | 1 |  |
|  | | | | | | | | | | | | | | | | | | | |

*Note.* Accuracy data filtered by 250 < RT < 1800 ms.

| Descriptive statistics of flanker's reaction times (RTs) by session, condition, age group, sex, and congruency. | | | | | | | | | | | | | | | | | | |
| --- | --- | --- | --- | --- | --- | --- | --- | --- | --- | --- | --- | --- | --- | --- | --- | --- | --- | --- |
|  | **Session** | | | **Condition** | | **Age Group** | **Sex** | | **Congruency** | | **Mean** | | **SD** | | **Minimum** | | **Maximum** | |
| **RTs** |  | 1 |  | Control |  | Older |  | Female |  | Congruent |  | 616.75 |  | 160.02 |  | 273 |  | 1577 |
|  |  |  |  |  |  |  |  |  |  | Incongruent |  | 694.48 |  | 160.16 |  | 426 |  | 1341 |
|  |  |  |  |  |  |  |  | Male |  | Congruent |  | 606.38 |  | 161.68 |  | 295 |  | 1620 |
|  |  |  |  |  |  |  |  |  |  | Incongruent |  | 641.75 |  | 139.30 |  | 333 |  | 1412 |
|  |  |  |  |  |  | Young |  | Female |  | Congruent |  | 460.21 |  | 124.04 |  | 261 |  | 1182 |
|  |  |  |  |  |  |  |  |  |  | Incongruent |  | 500.98 |  | 120.19 |  | 262 |  | 1150 |
|  |  |  |  |  |  |  |  | Male |  | Congruent |  | 450.91 |  | 105.18 |  | 250 |  | 1174 |
|  |  |  |  |  |  |  |  |  |  | Incongruent |  | 512.88 |  | 107.64 |  | 288 |  | 871 |
|  |  |  |  | Stress |  | Older |  | Female |  | Congruent |  | 541.88 |  | 117.41 |  | 334 |  | 1307 |
|  |  |  |  |  |  |  |  |  |  | Incongruent |  | 621.25 |  | 129.72 |  | 376 |  | 1361 |
| **RTs** |  | 1 |  | Stress |  | Older |  | Male |  | Congruent |  | 558.06 |  | 139.75 |  | 320 |  | 1165 |
|  |  |  |  |  |  |  |  |  |  | Incongruent |  | 608.88 |  | 140.00 |  | 343 |  | 1279 |
|  |  |  |  |  |  | Young |  | Female |  | Congruent |  | 427.10 |  | 95.56 |  | 269 |  | 837 |
|  |  |  |  |  |  |  |  |  |  | Incongruent |  | 475.63 |  | 105.77 |  | 264 |  | 815 |
|  |  |  |  |  |  |  |  | Male |  | Congruent |  | 443.69 |  | 88.49 |  | 288 |  | 895 |
|  |  |  |  |  |  |  |  |  |  | Incongruent |  | 488.92 |  | 99.41 |  | 309 |  | 987 |
|  |  | 2 |  | Control |  | Older |  | Female |  | Congruent |  | 541.40 |  | 129.60 |  | 324 |  | 1481 |
|  |  |  |  |  |  |  |  |  |  | Incongruent |  | 599.79 |  | 121.35 |  | 356 |  | 1577 |
|  |  |  |  |  |  |  |  | Male |  | Congruent |  | 504.07 |  | 122.61 |  | 300 |  | 937 |
|  |  |  |  |  |  |  |  |  |  | Incongruent |  | 565.17 |  | 129.24 |  | 342 |  | 1012 |
|  |  |  |  |  |  | Young |  | Female |  | Congruent |  | 410.78 |  | 90.99 |  | 269 |  | 858 |
|  |  |  |  |  |  |  |  |  |  | Incongruent |  | 466.57 |  | 96.35 |  | 251 |  | 802 |
|  |  |  |  |  |  |  |  | Male |  | Congruent |  | 433.47 |  | 114.48 |  | 291 |  | 1100 |
|  |  |  |  |  |  |  |  |  |  | Incongruent |  | 474.54 |  | 108.75 |  | 302 |  | 1277 |
|  |  |  |  | Stress |  | Older |  | Female |  | Congruent |  | 556.19 |  | 143.17 |  | 311 |  | 1405 |
|  |  |  |  |  |  |  |  |  |  | Incongruent |  | 622.75 |  | 136.43 |  | 316 |  | 1284 |
|  |  |  |  |  |  |  |  | Male |  | Congruent |  | 555.22 |  | 108.40 |  | 343 |  | 993 |
|  |  |  |  |  |  |  |  |  |  | Incongruent |  | 611.22 |  | 119.89 |  | 397 |  | 1108 |
|  |  |  |  |  |  | Young |  | Female |  | Congruent |  | 430.78 |  | 112.13 |  | 257 |  | 1322 |
|  |  |  |  |  |  |  |  |  |  | Incongruent |  | 467.03 |  | 105.46 |  | 259 |  | 1084 |
|  |  |  |  |  |  |  |  | Male |  | Congruent |  | 437.66 |  | 111.75 |  | 261 |  | 1161 |
|  |  |  |  |  |  |  |  |  |  | Incongruent |  | 480.42 |  | 95.61 |  | 257 |  | 945 |

*Note.* The table presents descriptives of correct RTs filtered by RT > 250, falling within ± 2.5 SD from the individual mean, and with one outlier participant removed (*N* = 99).

| **Mean Accuracy (Control: Congruent) Mean ACC (Control: Incongruent)**  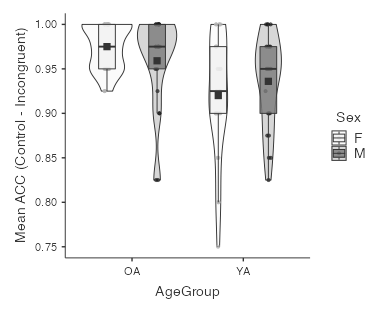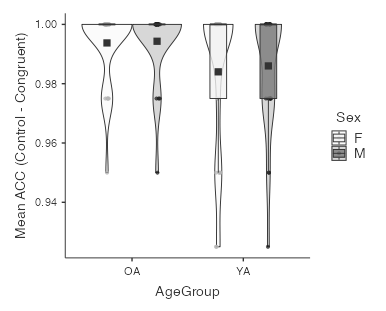  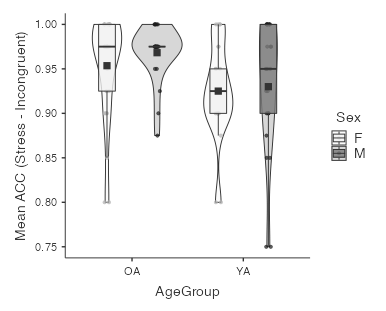 **Mean ACC (Stress: Congruent) Mean ACC (Stress: Incongruent)**  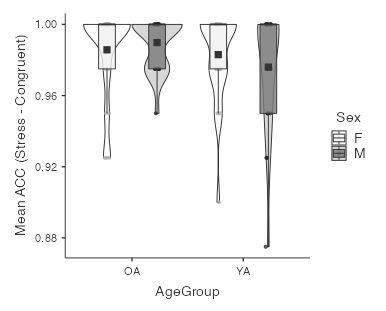**Mean RT (Control: Congruent) Mean RT (Control: Incongruent)**  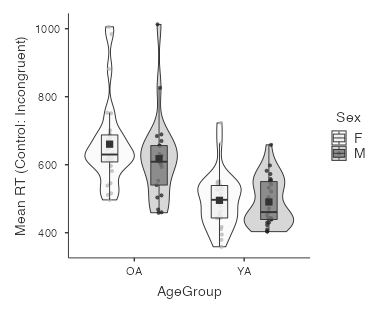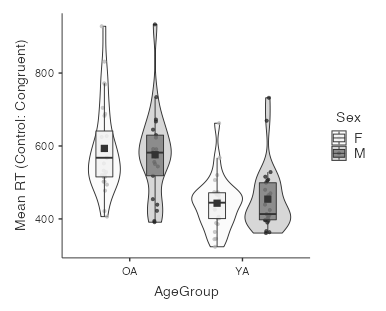  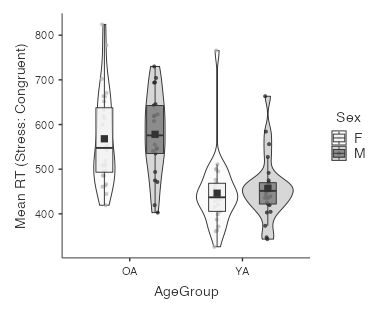**Mean RT (Stress: Congruent) Mean RT (Stress: Incongruent)**  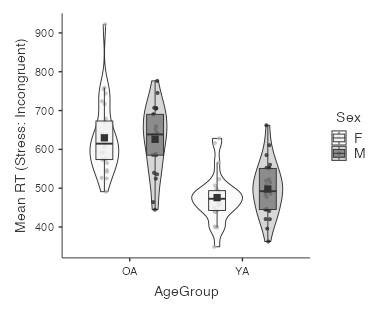 |
| --- |
|  |

# Flanker's accuracy model

- 1. Accuracy model information

| **Best-fitting Accuracy Model** | | | | | |
| --- | --- | --- | --- | --- | --- |
| **Info** | | **Value** | | **Comment** | |
| Model Type |  | Custom |  | Model with custom family |  |
| Call |  | glm |  | Accuracy ~ 1 + Condition + Age Group + Congruency + Session + Condition:Age Group + Condition:Congruency + Age Group:Congruency + Condition:Session + Age Group:Session + Congruency:Session + Condition:Age Group:Congruency + Condition:Age Group:Session + Condition:Congruency:Session + Age Group:Congruency:Session + Condition:Age Group:Congruency:Session + (1 + Congruency \| Subject) |  |
| Link function |  | Logit |  | Log of the odd of y=1 over y=0 |  |
| Distribution |  | Binomial |  | Dichotomous event distribution of y |  |
| LogLikel. |  | -1940.32 |  | Unconditional Log-Likelihood |  |
| -2*LogLikel. |  | 3880.65 |  | Unconditional absolute deviance |  |
| Deviance |  | 3686.71 |  | Conditional relative deviance |  |
| R-squared |  | 0.18 |  | Marginal |  |
| R-squared |  | 0.28 |  | Conditional |  |
| AIC |  | 3918.65 |  | Less is better |  |
| BIC |  | 4063.49 |  | Less is better |  |
| Residual DF |  | 15096.00 |  |  |  |
| Chi-squared/DF |  | 0.86 |  | Overdispersion indicator |  |
| Converged |  | yes |  |  |  |
| Optimizer |  | bobyqa |  |  |  |
|  | | | | | |

- 1. Accuracy model results

| Fixed Effect Omnibus tests | | | | | | | |
| --- | --- | --- | --- | --- | --- | --- | --- |
|  | | **X²** | | **df** | | **p** | |
| Condition |  | 9.34 |  | 1.00 |  | 0.002 |  |
| Age Group |  | 20.20 |  | 1.00 |  | < .001 |  |
| Congruency |  | 67.47 |  | 1.00 |  | < .001 |  |
| Session |  | 4.43 |  | 1.00 |  | 0.035 |  |
| Condition ✻ Age Group |  | 3.06 |  | 1.00 |  | 0.080 |  |
| Condition ✻ Congruency |  | 2.99 |  | 1.00 |  | 0.084 |  |
| Age Group ✻ Congruency |  | 0.02 |  | 1.00 |  | 0.892 |  |
| Condition ✻ Session |  | 0.01 |  | 1.00 |  | 0.908 |  |
| Age Group ✻ Session |  | 0.08 |  | 1.00 |  | 0.783 |  |
| Congruency ✻ Session |  | 0.39 |  | 1.00 |  | 0.534 |  |
| Condition ✻ Age Group ✻ Congruency |  | 0.75 |  | 1.00 |  | 0.387 |  |
| Condition ✻ Age Group ✻ Session |  | 0.87 |  | 1.00 |  | 0.351 |  |
| Condition ✻ Congruency ✻ Session |  | 0.27 |  | 1.00 |  | 0.605 |  |
| Age Group ✻ Congruency ✻ Session |  | 0.42 |  | 1.00 |  | 0.516 |  |
| Condition ✻ Age Group ✻ Congruency ✻ Session |  | 0.89 |  | 1.00 |  | 0.347 |  |
|  | | | | | | | |

# Flanker's RTs model

- 1. RTs model information

| **Best-fitting RT Model** | | | | | |
| --- | --- | --- | --- | --- | --- |
| **Info** | | **Value** | | **Comment** | |
| Model Type |  | Custom |  | Model with custom family |  |
| Call |  | glm |  | RT ~ 1 + Condition + Age Group + Congruency + Session + Condition:Age Group + Condition:Congruency + Age Group:Congruency + Condition:Session + Age Group:Session + Congruency:Session + Condition:Age Group:Congruency + Condition:Age Group:Session + Condition:Congruency:Session + Age Group:Congruency:Session + Condition:Age Group:Congruency:Session + (1 + Condition \| Subject) |  |
| Link function |  | Identity |  | Coefficients in the same scale of y |  |
| Distribution |  | . |  | . |  |
| LogLikel. |  | -83302.73 |  | Unconditional Log-Likelihood |  |
| -2*LogLikel. |  | 166605.46 |  | Unconditional absolute deviance |  |
| Deviance |  | 0.76 |  | Conditional relative deviance |  |
| R-squared |  | NaN |  | Marginal |  |
| R-squared |  | NaN |  | Conditional |  |
| AIC |  | 166645.46 |  | Less is better |  |
| BIC |  | 166796.66 |  | Less is better |  |
| Residual DF |  | 14168.00 |  |  |  |
| Chi-squared/DF |  | 0.00 |  | Overdispersion indicator |  |
| Converged |  | yes |  |  |  |
| Optimizer |  | bobyqa |  |  |  |
| Note. *R^2^* cannot be computed. | | | | | |

- 1. RTs model results

| Fixed Effect Omnibus tests | | | | | | | |
| --- | --- | --- | --- | --- | --- | --- | --- |
|  | | **X²** | | **df** | | **p** | |
| Condition |  | 5.80 |  | 1.00 |  | 0.016 |  |
| Age Group |  | 438.73 |  | 1.00 |  | < .001 |  |
| Congruency |  | 1173.63 |  | 1.00 |  | < .001 |  |
| Session |  | 63.92 |  | 1.00 |  | < .001 |  |
| Condition ✻ Age Group |  | 1.62 |  | 1.00 |  | 0.203 |  |
| Condition ✻ Congruency |  | 1.12 |  | 1.00 |  | 0.291 |  |
| Age Group ✻ Congruency |  | 41.55 |  | 1.00 |  | < .001 |  |
| Condition ✻ Session |  | 19.56 |  | 1.00 |  | < .001 |  |
| Age Group ✻ Session |  | 7.41 |  | 1.00 |  | 0.006 |  |
| Congruency ✻ Session |  | 0.22 |  | 1.00 |  | 0.637 |  |
| Condition ✻ Age Group ✻ Congruency |  | 1.55 |  | 1.00 |  | 0.213 |  |
| Condition ✻ Age Group ✻ Session |  | 7.24 |  | 1.00 |  | 0.007 |  |
| Condition ✻ Congruency ✻ Session |  | 0.11 |  | 1.00 |  | 0.735 |  |
| Age Group ✻ Congruency ✻ Session |  | 0.64 |  | 1.00 |  | 0.423 |  |
| Condition ✻ Age Group ✻ Congruency ✻ Session |  | 0.00 |  | 1.00 |  | 0.947 |  |
|  | | | | | | | |

# Cortisol model

| Model Info | | | |
| --- | --- | --- | --- |
|  |  |  |  |
| **Info** | |  | |
| Estimate |  | Linear mixed model fit by ML |  |
| Call |  | CORT.log10 ~ 1 + condition + age + sex + time + condition:age + condition:sex + age:sex + condition:time + age:time + sex:time + condition:age:time+( 1 \| ID ) |  |
| AIC |  | -1069.40 |  |
| BIC |  | -832.97 |  |
| LogLikel. |  | 578.70 |  |
| R-squared Marginal |  | 0.36 |  |
| R-squared Conditional |  | 0.71 |  |
| Converged |  | yes |  |
| Optimizer |  | bobyqa |  |
|  | | | |

**Model Results**

| Fixed Effect Omnibus tests | | | | | | | | | |
| --- | --- | --- | --- | --- | --- | --- | --- | --- | --- |
|  |  |  |  |  |  |  |  |  |  |
|  | | ***F*** | | **Num df** | | **Den df** | | ***p*** | |
| condition |  | 285.31 |  | 1 |  | 1493.06 |  | < .001 |  |
| age |  | 15.86 |  | 1 |  | 100.00 |  | < .001 |  |
| sex |  | 26.87 |  | 1 |  | 100.00 |  | < .001 |  |
| time |  | 26.58 |  | 7 |  | 1493.06 |  | < .001 |  |
| condition ✻ age |  | 17.77 |  | 1 |  | 1493.06 |  | < .001 |  |
| condition ✻ sex |  | 8.88 |  | 1 |  | 1493.06 |  | 0.003 |  |
| age ✻ sex |  | 10.95 |  | 1 |  | 100.00 |  | 0.001 |  |
| condition ✻ time |  | 20.93 |  | 7 |  | 1493.03 |  | < .001 |  |
| age ✻ time |  | 8.87 |  | 7 |  | 1493.06 |  | < .001 |  |
| sex ✻ time |  | 2.73 |  | 7 |  | 1493.06 |  | 0.008 |  |
| condition ✻ age ✻ time |  | 2.35 |  | 7 |  | 1493.03 |  | 0.022 |  |
| Note. Satterthwaite method for degrees of freedom | | | | | | | | | |
|  | | | | | | | | | |

**Simple Effects: Condition (per level of age and time)**

| Simple effects of condition : Parameter estimates | | | | | | | | | | | | | | | | | | | |
| --- | --- | --- | --- | --- | --- | --- | --- | --- | --- | --- | --- | --- | --- | --- | --- | --- | --- | --- | --- |
| **Moderator levels** | | | |  | | | | | | **95% Confidence Interval** | | | |  | | | | | |
| **age** | | **time** | | **contrast** | | **Estimate** | | **SE** | | **Lower** | | **Upper** | | **df** | | ***t*** | | ***p*** | |
| Older |  | 0 |  | Stress - Control |  | -0.01 |  | 0.03 |  | -0.07 |  | 0.05 |  | 1493.00 |  | -0.24 |  | 0.814 |  |
|  |  | 10 |  | Stress - Control |  | 0.01 |  | 0.03 |  | -0.05 |  | 0.07 |  | 1493.00 |  | 0.45 |  | 0.654 |  |
|  |  | 20 |  | Stress - Control |  | 0.07 |  | 0.03 |  | 0.01 |  | 0.13 |  | 1493.00 |  | 2.43 |  | 0.015 |  |
|  |  | 30 |  | Stress - Control |  | 0.15 |  | 0.03 |  | 0.09 |  | 0.21 |  | 1493.00 |  | 4.83 |  | < .001 |  |
|  |  | 40 |  | Stress - Control |  | 0.17 |  | 0.03 |  | 0.11 |  | 0.23 |  | 1493.22 |  | 5.56 |  | < .001 |  |
|  |  | 50 |  | Stress - Control |  | 0.16 |  | 0.03 |  | 0.10 |  | 0.22 |  | 1493.12 |  | 5.18 |  | < .001 |  |
|  |  | 60 |  | Stress - Control |  | 0.13 |  | 0.03 |  | 0.07 |  | 0.19 |  | 1493.10 |  | 4.35 |  | < .001 |  |
|  |  | 75 |  | Stress - Control |  | 0.09 |  | 0.03 |  | 0.03 |  | 0.15 |  | 1493.13 |  | 2.78 |  | 0.005 |  |
| Young |  | 0 |  | Stress - Control |  | -0.03 |  | 0.03 |  | -0.09 |  | 0.03 |  | 1493.00 |  | -1.13 |  | 0.260 |  |
|  |  | 10 |  | Stress - Control |  | -0.02 |  | 0.03 |  | -0.08 |  | 0.04 |  | 1493.00 |  | -0.79 |  | 0.427 |  |
|  |  | 20 |  | Stress - Control |  | 0.12 |  | 0.03 |  | 0.06 |  | 0.18 |  | 1493.00 |  | 4.00 |  | < .001 |  |
|  |  | 30 |  | Stress - Control |  | 0.29 |  | 0.03 |  | 0.23 |  | 0.35 |  | 1493.00 |  | 9.56 |  | < .001 |  |
|  |  | 40 |  | Stress - Control |  | 0.29 |  | 0.03 |  | 0.23 |  | 0.35 |  | 1493.00 |  | 9.62 |  | < .001 |  |
|  |  | 50 |  | Stress - Control |  | 0.25 |  | 0.03 |  | 0.19 |  | 0.31 |  | 1493.00 |  | 8.31 |  | < .001 |  |
|  |  | 60 |  | Stress - Control |  | 0.22 |  | 0.03 |  | 0.16 |  | 0.28 |  | 1493.00 |  | 7.18 |  | < .001 |  |
|  |  | 75 |  | Stress - Control |  | 0.17 |  | 0.03 |  | 0.11 |  | 0.23 |  | 1493.00 |  | 5.70 |  | < .001 |  |
| Note. Simple effects are estimated keeping constant other independent variable(s) in the model | | | | | | | | | | | | | | | | | | | |

# HR model

| Model Info | | | |
| --- | --- | --- | --- |
|  |  |  |  |
| **Info** | |  | |
| Estimate |  | Linear mixed model fit by ML |  |
| Call |  | HR ~ 1 + condition + age + sex + time + condition:age + age:sex + condition:time+( 1 \| ID ) |  |
| AIC |  | 6867.90 |  |
| BIC |  | 6945.97 |  |
| LogLikel. |  | -3417.95 |  |
| R-squared Marginal |  | 0.22 |  |
| R-squared Conditional |  | 0.67 |  |
| Converged |  | yes |  |
| Optimizer |  | bobyqa |  |
|  | | | |

**Model Results**

| Fixed Effect Omnibus tests | | | | | | | | | |
| --- | --- | --- | --- | --- | --- | --- | --- | --- | --- |
|  |  |  |  |  |  |  |  |  |  |
|  | | ***F*** | | **Num df** | | **Den df** | | ***p*** | |
| condition |  | 58.47 |  | 1 |  | 876.97 |  | < .001 |  |
| age |  | 6.28 |  | 1 |  | 100.28 |  | 0.014 |  |
| sex |  | 1.43 |  | 1 |  | 100.28 |  | 0.235 |  |
| time |  | 82.90 |  | 4 |  | 872.38 |  | < .001 |  |
| condition ✻ age |  | 13.38 |  | 1 |  | 876.97 |  | < .001 |  |
| age ✻ sex |  | 6.21 |  | 1 |  | 100.28 |  | 0.014 |  |
| condition ✻ time |  | 12.19 |  | 4 |  | 872.38 |  | < .001 |  |
| Note. Satterthwaite method for degrees of freedom | | | | | | | | | |
|  | | | | | | | | | |

**Simple Effects : Condition (per level of time)**

| Simple effects of condition : Parameter estimates | | | | | | | | | | | | | | | | | |  |
| --- | --- | --- | --- | --- | --- | --- | --- | --- | --- | --- | --- | --- | --- | --- | --- | --- | --- | --- |
| **Moderator levels** | |  | | | | | | **95% Confidence Interval** | | | |  | | | | | |  |
| **time** | | **contrast** | | **Estimate** | | **SE** | | **Lower** | | **Upper** | | **df** | | ***t*** | ***p*** | | |  |
| Baseline |  | Stress - Control |  | -0.58 |  | 1.02 |  | -2.58 |  | 1.43 |  | 873.32 |  | -0.56 |  | 0.574 |  | |
| Ant. |  | Stress - Control |  | 3.09 |  | 1.02 |  | 1.09 |  | 5.09 |  | 873.23 |  | 3.03 |  | 0.003 |  | |
| TSST/Control |  | Stress - Control |  | 9.10 |  | 1.03 |  | 7.09 |  | 11.12 |  | 873.42 |  | 8.87 |  | < .001 |  | |
| Post |  | Stress - Control |  | 4.09 |  | 1.02 |  | 2.09 |  | 6.10 |  | 873.30 |  | 4.01 |  | < .001 |  | |
| Post2 |  | Stress - Control |  | 1.91 |  | 1.02 |  | -0.10 |  | 3.91 |  | 873.30 |  | 1.87 |  | 0.062 |  | |
| Note. Simple effects are estimated keeping constant other independent variable(s) in the model | | | | | | | | | | | | | | | | | |  |

# Subjective stress model

| Model Info | | | |
| --- | --- | --- | --- |
| **Info** | |  | |
| Estimate |  | Linear mixed model fit by ML |  |
| Call |  | VAS ~ 1 + condition + age + time + sex + condition:age + condition:time + age:time+( 1 \| ID ) |  |
| AIC |  | 10056.43 |  |
| BIC |  | 10168.41 |  |
| LogLikel. |  | -5006.21 |  |
| R-squared Marginal |  | 0.09 |  |
| R-squared Conditional |  | 0.62 |  |
| Converged |  | yes |  |
| Optimizer |  | bobyqa |  |
|  | | | |

**Model Results**

| Fixed Effect Omnibus tests | | | | | | | | | |
| --- | --- | --- | --- | --- | --- | --- | --- | --- | --- |
|  | | ***F*** | | **Num df** | | **Den df** | | ***p*** | |
| condition |  | 98.92 |  | 1 |  | 1100.00 |  | < .001 |  |
| age |  | 0.60 |  | 1 |  | 100.00 |  | 0.441 |  |
| time |  | 16.54 |  | 5 |  | 1100.00 |  | < .001 |  |
| sex |  | 0.23 |  | 1 |  | 100.00 |  | 0.630 |  |
| condition ✻ age |  | 5.23 |  | 1 |  | 1100.00 |  | 0.022 |  |
| condition ✻ time |  | 11.50 |  | 5 |  | 1100.00 |  | < .001 |  |
| age ✻ time |  | 2.73 |  | 5 |  | 1100.00 |  | 0.018 |  |
| Note. Satterthwaite method for degrees of freedom | | | | | | | | | |
|  | | | | | | | | | |

## Simple Effects : Condition (per level of time)

| Simple effects of condition : Parameter estimates | | | | | | | | | | | | | | | | | |  |
| --- | --- | --- | --- | --- | --- | --- | --- | --- | --- | --- | --- | --- | --- | --- | --- | --- | --- | --- |
| **Moderator levels** | |  | | | | | | **95% Confidence Interval** | | | |  | | | | | |  |
| **time** | | **contrast** | | **Estimate** | | **SE** | | **Lower** | | **Upper** | | **df** | | ***t*** | | ***p*** | |  |
| 0 |  | Stress - Control |  | -0.24 |  | 1.97 |  | -4.10 |  | 3.62 |  | 1100.00 |  | -0.12 |  | 0.903 |  | |
| 10 |  | Stress - Control |  | 12.97 |  | 1.97 |  | 9.11 |  | 16.83 |  | 1100.00 |  | 6.59 |  | < .001 |  | |
| 20 |  | Stress - Control |  | 17.56 |  | 1.97 |  | 13.70 |  | 21.42 |  | 1100.00 |  | 8.92 |  | < .001 |  | |
| 30 |  | Stress - Control |  | 9.85 |  | 1.97 |  | 5.99 |  | 13.71 |  | 1100.00 |  | 5.01 |  | < .001 |  | |
| 40 |  | Stress - Control |  | 4.82 |  | 1.97 |  | 0.96 |  | 8.68 |  | 1100.00 |  | 2.45 |  | 0.014 |  | |
| 50 |  | Stress - Control |  | 2.98 |  | 1.97 |  | -0.88 |  | 6.84 |  | 1100.00 |  | 1.51 |  | 0.130 |  | |
| Note. Simple effects are estimated keeping constant other independent variable(s) in the model | | | | | | | | | | | | | | | | | |  |
|  | | | | | | | | | | | | | | | | | |  |
